# Low-density lipoprotein receptor-related protein 8 facilitates the proliferation and invasion of non-small cell lung cancer cells by regulating the Wnt/β-catenin signaling pathway

**DOI:** 10.1080/21655979.2022.2036917

**Published:** 2022-03-04

**Authors:** Zhi Fang, Min Zhong, Ling Zhou, Yi Le, Heng Wang, Ziling Fang

**Affiliations:** a Department of Oncology, The First Affiliated Hospital of Nanchang University, Nanchang, People’s Republic of China; b Department of Abdominal Oncology, Jiangxi Key Laboratory for Individualized Cancer Therapy, Nanchang, People’s Republic of China; c Department of Orthopedics, The First Affiliated Hospital of Nanchang University, Nanchang, People’s Republic of China

**Keywords:** LRP8, NSCLC, Wnt/β-catenin, proliferation, invasion

## Abstract

Low-density lipoprotein receptor-related protein 8 (LRP8) is involved in the development of multiple tumors, including lung cancer. However, the exact mechanism by which LRP8 exerts its oncogenic role in non-small cell lung cancer (NSCLC) remains elusive. Hence, in this study, we aimed to unravel the expression and role of LRP8 in the progression of NSCLC. We used online bioinformatics databases to identify the expression of LRP8 in multiple types of lung cancer. We validated LRP8 expression in NSCLC cell lines and tissues by Western blotting and immunohistochemistry. The functions of LRP8 in NSCLC carcinogenesis and progression were determined using *in vitro* and *in vivo* systems. The Wnt pathway activator LiCl was further used to validate the regulatory role of LRP8 in Wnt/β-catenin signaling. We demonstrated that LRP8 was markedly overexpressed in NSCLC tissues and cell lines, and its overexpression significantly correlated with poor clinicopathological characteristics and prognosis. Moreover, LRP8 depletion suppressed cell proliferation, migration, invasion, and epithelial-mesenchymal transition *in vitro* and impeded tumor growth *in vivo*. Mechanistically, LPR8 knockdown elicited tumor-suppressive functions by suppressing the Wnt/β-catenin pathway, which was partially reversed by LiCl. Hence, our study revealed that LRP8 facilitates NSCLC cell proliferation and invasion via the Wnt/β-catenin pathway, and thus LRP8 could be a novel therapeutic target for NSCLC.

## Graphical abstract

**Figure:**
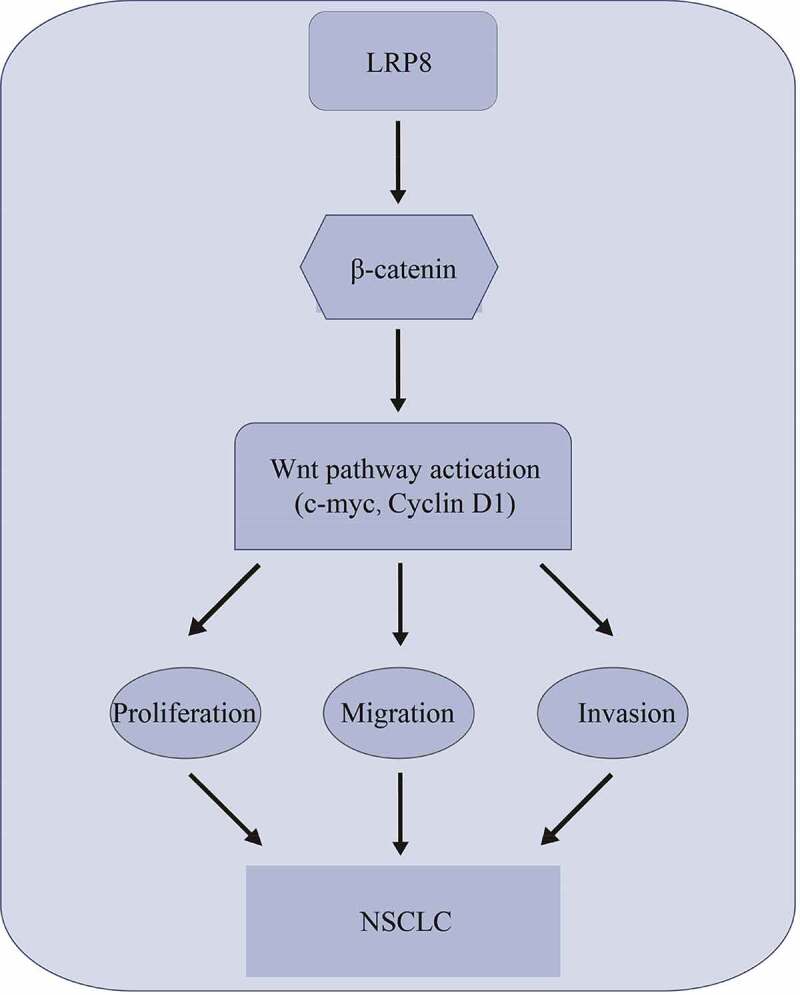

## Introduction

Non-small cell lung cancer (NSCLC) is among the most common cancers worldwide. It accounts for 85% of lung cancers and has led to millions of cancer-related deaths [1]. Despite significant advancements in the diagnosis and treatment of NSCLC over the past two decades, the overall survival rates are still low, especially in patients in whom the cancer has metastasized [2,3]. Therefore, it is imperative to explore promising novel targets to improve the survival of patients with NSCLC.

Low-density lipoprotein receptor-related protein 8 (LRP8), also known as apolipoprotein E receptor 2 (ApoER2), is a member of the low-density lipoprotein receptor (LDLR) family and is involved in signal transduction and specific ligand endocytosis [4]. Since its first identification in the brain regulating the cholesterol transport protein apolipoprotein E (ApoE), LRP8 has been found to be involved in numerous pathological or physiological processes [5–7]. In addition, LRP8 promotes the tumorigenesis and progression of several cancers, such as melanoma, gastric cancer, and prostate cancer [8–12]. Via a genome-wide CRISPR activation screening, Cai et al. [13] found that LRP8 is responsible for the resistance of hepatocellular carcinoma cells to sorafenib. Moreover, the ApoE2-LRP8 axis is critical for modulating pancreatic cancer cell proliferation and cell cycle progression [14]. However, the function and mechanism of LRP8 in NSCLC remain unknown.

The Wnt signal transduction pathway is a canonical pathway whose aberration in human tumor cells makes the cells susceptible to metastasis and adhesion by mediating the epithelial-to-mesenchymal transition (EMT) [15,16]. Based on the canonical Wnt signaling pathway hypothesis, the pathway is activated only when β-catenin accumulates and translocates to the nucleus after receiving a Wnt ligand stimulation, which forms a complex with LRP [17]. Alterations in the Wnt/β-catenin pathway contribute to multiple cancers, including osteosarcoma and NSCLC [18,19]. LRP8 also modulates osteoblast differentiation and mineralization by diminishing Wnt pathway activation [20]. Moreover, Lin et al. reported that deletion of LRP8 sensitizes triple negative breast cancer cells to chemotherapy [21]. However, whether LRP8 has a cancer-promoting role in NSCLC via Wnt/β-catenin signaling has not been reported.

In the present study, we aimed to understand whether LRP8 is involved in NSCLC and the mechanism behind its function. We found a high expression of LRP8 in NSCLC cells and tissues, and its overexpression was associated with poor prognosis in NSCLC patients. Moreover, LRP8 promoted the proliferation, migration, invasion, and EMT of NSCLC cells. Mechanistically, LRP8 accelerated the progression of NSCLC by modulating the Wnt/β-catenin signaling pathway. We believe that targeting LRP8 may be a new avenue to prevent the development of NSCLC.

## Materials and methods

### Tissue specimens

Sixty paired paraffin-embedded NSCLC tissues and adjacent normal tissues were obtained from patients with primary NSCLC diagnosed between June 2014 and June 2018 at the First Affiliated Hospital of Nanchang University (Nanchang, China). The clinical information of the patients is summarized in Table 1. This study has been approved by the Medical Research Ethics Committee of the First Affiliated Hospital of Nanchang University. All patients who participated in this study signed the written informed consent form.

**Table 1.** The relationship between LRP8 and clinical factors of NSCLC patients

		LRP8 expression		
Parameters	n = 60	Low (18)	High (42)	*p*-value
Gender				
Male	30	11	19	0.260
Female	30	7	23	
Age(y)				
≤60	34	10	24	0.909
>60	26	8	18	
Differentiation				
Poor	35	9	26	0.391
Moderate/well	25	9	16	
Tumor size (cm)				
≤4	23	11	12	**0.018**
>4	37	7	30	
TNM stage				
I + II	31	13	18	**0.037**
III + IV	29	5	24	
T stage				
pT1 + pT2	40	16	24	**0.019**
pT3 + pT4	20	2	18	
Lymph node status				
N0	25	12	13	**0.010**
N1 + N2+ N3	35	6	29	
Total	60	18	42	

### Immunohistochemistry (IHC)

IHC assay was performed as previously described [22]. Briefly, the paraffin-embedded sections were deparaffinized with xylene for 30 min and rehydrated in graded ethanol. Subsequently, antigen retrieval was performed using citrate buffer (pH 6.0) at a high temperature. The sections were blocked with goat serum at 37°C for 1 h, followed by incubation with primary antibody against LRP8 (Abclonal, China) at dilution of 1:200 at 4°C overnight and goat anti-rabbit secondary antibody (Abcam, USA) for 30 min at 37°C. The sections were then stained with diaminobenzobutyl and hematoxylin. All the sections were blindly and independently evaluated and scored by two experienced pathologists. The staining index criteria were based on a previous study [23].

### Cell lines and cell culture

The cell lines used in our study, including human NSCLC cell lines (95-D, H1299, H460, HCC-827, A549, PC-9, and H1975) and human normal bronchial epithelioid cells (HBE) (Procell Life Science and Technology, Wuhan, China), were cultured in Roswell Park Memorial Institute 1640 (RPMI-1640) medium containing 10% fetal bovine serum (Biological, Kibbutz Beit Haemek, Israel) with penicillin and streptomycin and incubated in an environment with 5% CO_2_ at 37°C.

### Cell transfection

To knock down LRP8 in NSCLC cells, two small interfering RNAs (siRNAs), scrambled short-hairpin RNA (shRNA) and matched negative controls purchased from GenePharm (Shanghai, China) were transfected into NSCLC cells. The LRP8 overexpression plasmid or an empty vector (GenePharm) was used to upregulate the expression of LRP8. The NSCLC cells were seeded in 6-well plates and transfected with the plasmids via the TurboFect transfection reagent (Thermo Fischer Scientific, USA) when the cell confluency reached 50–70%. After incubation for 48 h, the cells were harvested for further experiments. The sequences of the LRP8 siRNAs used in this study were as follows: LRP8-Homo-1082, 5′-CGCGACUGCAAAGACAAAUTT-3′; LRP8-Homo-1642, 5′-CUCCUACCGUAAGAUCUAUTT-3′.

### Western blotting analysis

The tissues and cells were subjected to protein isolation after lysis in RIPA buffer (Applygen Tech Inc., China) containing 1% protease/phosphatase inhibitor. Then, a 10% ExpressCast PAGE Gel Preparation Kit (New Cell and Molecular Biotech, China) was used to separate the proteins. The polyvinylidene difluoride membranes were used for proteins transfer, and the membranes were then blocked with 5% milk. Subsequently, antibodies against LRP8 (NB100-2216, Novus Biologicals, USA), GAPDH (60,004-1-Ig; Proteintech, USA), β-catenin (8480s, Cell Signaling Technology, USA), c-Myc (5605s, Cell Signaling Technology), cyclin D1 (2978s, Cell Signaling Technology), E-cadherin (ab1416; Abcam), N-cadherin (ab98952, Abcam), and vimentin (5741s, Cell Signaling Technology) were used to incubate the membranes overnight. The bands were observed and analyzed using the chemiluminescence reagent (Proteintech) after 1 h incubation with the corresponding secondary antibodies (Proteintech).

### Cell counting Kit-8 (CCK8) assay

Briefly, the transfected cells were seeded into a 96-well plate at a density of 2 × 10^3^ cells per well. Then, 10 µL CCK8 solution (Glpbio, California, USA) was added into the wells with 100 µL of complete medium and incubated for 30 min. The proliferation of NSCLC cells was measured and evaluated at 450 nm for four consecutive days.

### Colony formation assay

At 48 h after transfection, approximately 500 cells were seeded into 6-well plates and cultured in a fresh medium for 12 days. Then, 4% paraformaldehyde and crystal violet were used to fix and stain the colonies, respectively. Finally, the colonies were quantified.

### Transwell assay

Transwell migration and invasion assays were carried out as previously described [24]. After transfection with si-LRP8 or LRP8 plasmid for 48 h, the collected cells were resuspended in a serum-free medium. Then, 200 µL serum-free medium containing 3 × 10^4^ cells were added to the transwell upper chamber coated with 60 µL Matrigel (Corning, USA) for the invasion assay and without the Matrigel for the migration assay. The lower chamber was filled with 600 µL of complete medium for both assays. After incubation for 24 h, the cells that invaded or migrated to the bottom surface of the upper compartment were fixed and stained with 4% paraformaldehyde and crystal violet, respectively. The cells were imaged and counted under a microscope.

### Mouse xenograft tumor growth assay

Ten five-week-old female nude mice purchased from Hangzhou Ziyuan Laboratory Animal Technology Company (Hangzhou, China) were randomly divided into two groups. The H460 cells transfected with the lentiviral vector containing LRP8 shRNA (Lv-sh-LRP8) or negative control (Lv-sh-Con) (Genechem, China) were screened using puromycin to obtain stable cell lines. The assay was performed as described in our previous study [25]. We subcutaneously injected 1 × 10^7^ steady H460 cells of sh-LRP8 and sh-Con groups into the left axillary skin of nude mice. The volume and weight of the tumors in the two groups were measured and recorded every week for four consecutive weeks. The volume was calculated as follows: volume = (length × width^2^)/2 (mm^3^). The mice were euthanized, and the tumors were harvested for further study. All experiments involving animals were approved by the institutional research ethics committee.

### Statistical analysis

All experiments involved in our study were repeated at least thrice. The data were analyzed using the SPSS software (version 26.0; SPSS Inc., Chicago, IL, USA) and presented as mean ± standard deviation (SD). The differences between groups were distinguished by the two-tailed Student’s t-test or the χ^2^ test. The Kaplan–Meier method was used to confirm the overall survival rate of NSCLC patients recruited in our study. Statistical significance was set at *p* < 0.05.

## Result

### LRP8 is associated with the poor prognosis of NSCLC patients

**Figure 1.**
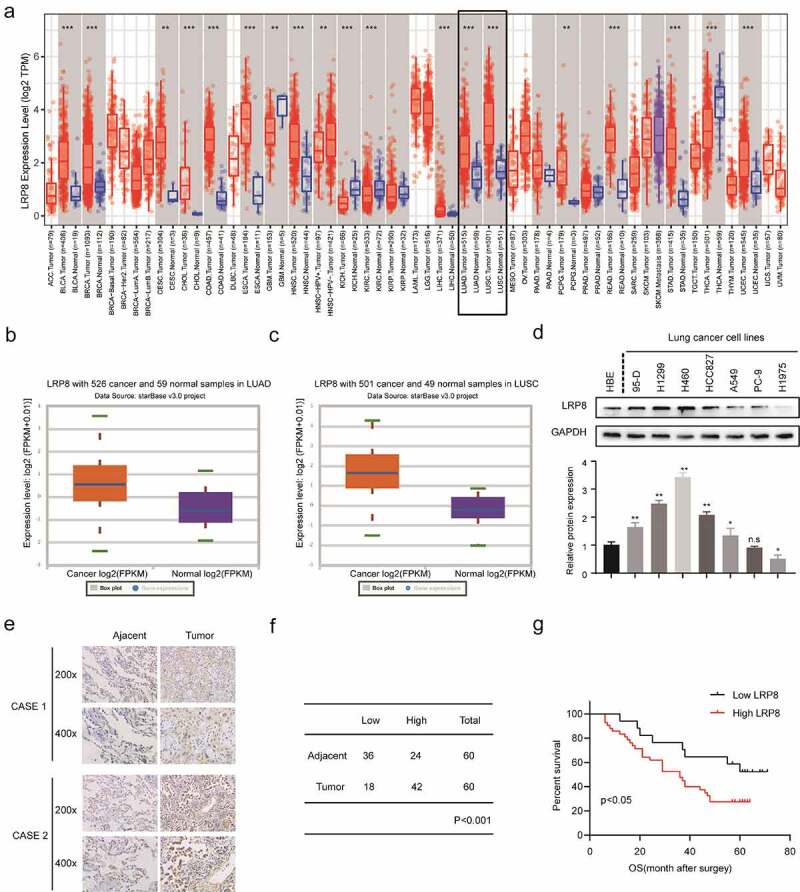
LRP8 is related to the poor prognosis of NSCLC patients. (a–c) Expression of LRP8 in NSCLC tissues as analyzed by TIMER and StarBase3.0 databases. (d) Western blotting assays to detect the expression of LRP8 in seven NSCLC cell lines and the normal bronchial epithelioid cells. (e) Immunohistochemistry staining of two representative cases showing the expression and location of LRP8 in NSCLC tissues. (f) Four-grid table showing the statistical difference of LRP8 level between tumor tissues and normal adjacent tissues. (g) Kaplan–Meier curve based on LRP8 expression in 60 NSCLC patients (log-rank test, p < 0.05). n.s, no significant difference, *p < 0.05, **p < 0.01. At least three independent biological experiments were repeated, and the data were presented as mean ± SD.

The expression of LRP8 in the two most prevalent types of NSCLC, lung adenocarcinoma (LUAD) and lung squamous cell carcinoma (LUSC), and adjacent normal lung tissues were evaluated by bioinformatics analysis [26]. The Tumor IMmune Estimation Resource (TIMER) database (https://cistrome.shinyapps.io/timer/) showed that LRP8 was highly expressed in various tumor tissues, including NSCLC (Figure 1(a)) [27]. Similarly, data obtained from StarBase3.0 (http://starbase.sysu.edu.cn/) revealed that LRP8 was elevated in LUAD and LUSC compared to normal tissues (Figures 1(b) and (c)) [28]. Subsequently, IHC analysis showed that the expression of LRP8 was remarkably higher in most of the NSCLC cell lines, especially in H460 and H1299 than in HBE normal cells (Figure 1(d)). In addition, IHC analysis of the 60 paired NSCLC tissues and their adjacent normal tissues demonstrated a high level of LRP8 in the cytoplasm of the NSCLC tissues than that of the non-cancerous tissues (Figure 1(e)). We classified 70% (42/60) of NSCLC tissues and 40% (24/60) of paired normal tissues as having high LRP8 expression (*p* < 0.001, Figure 1(f)). Furthermore, the Kaplan–Meier curve analysis revealed that the increased expression of LRP8 was related to a poorer prognosis in patients with NSCLC (Figure 1(g)).

### LRP8 promoted NSCLC proliferation in vitro

**Figure 2.**
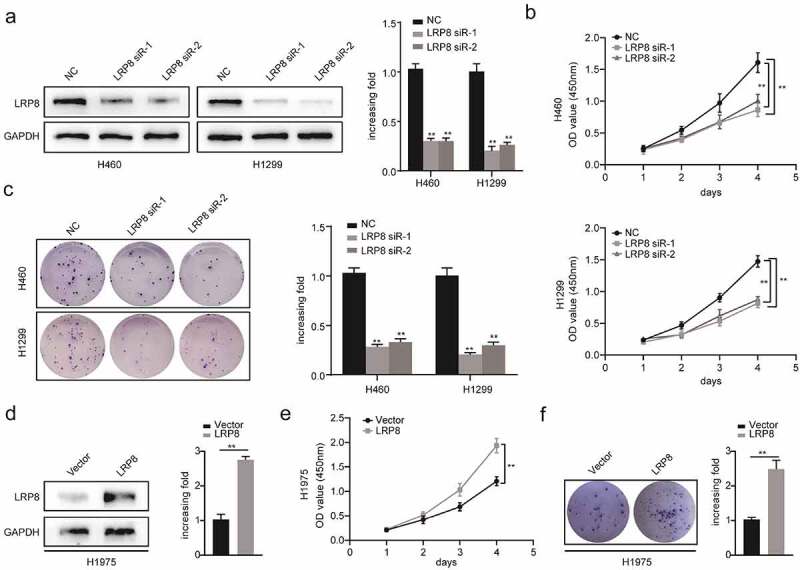
LRP8 promoted NSCLC proliferation *in vitro*. Western blotting experiments were conducted to validate the transfection efficiency of LRP8 siRNA in H460 and H1299 (a) and LRP8 overexpression plasmid in H1975 (d). CCK-8 assay was used to evaluate the proliferation ability of H460 and H1299 cells transfected with LRP8 siRNAs (b) and H1975 cells with LRP8 plasmid (e). (c) Colony formation analysis showing differences in H1299 and H460 cell proliferation among the three groups. (f) H1975 cell viability was measured using colony formation analysis. **p < 0.01. All experiments were performed independently at least three times, and the results were presented as mean ± SD.

To explore the role of LRP8 in NSCLC cell viability *in vitro*, we performed the CCK8 and colony formation assays. After transfecting plasmids into NSCLC cells, the plasmids showed a high transfection efficiency, as reflected by a significantly decreased LRP8 expression in the LRP8 siRNA groups in H1299 and H460 cells, and significantly increased LRP8 expression in the LRP8 overexpression group in H1975 cells (Figures 2(a) and (d)). The CCK8 assay showed that silencing LRP8 lowered the cell viability in both H1299 and H460 cells (Figure 2(b)). In contrast, LRP8 overexpression in H1975 cells promoted proliferation (Figure 2(e)). Similarly, colony formation assays revealed that the number of colonies significantly reduced in H460 and H1299 cells transfected with LRP8 siRNA and significantly increased in H1975 cells transfected with the LRP8 plasmid compared to the corresponding negative control group (Figures 2(c) and (f)). In summary, the expression of LRP8 positively correlated with NSCLC cell proliferation *in vitro*.

### LRP8 enhanced the metastasis of NSCLC cells

**Figure 3.**
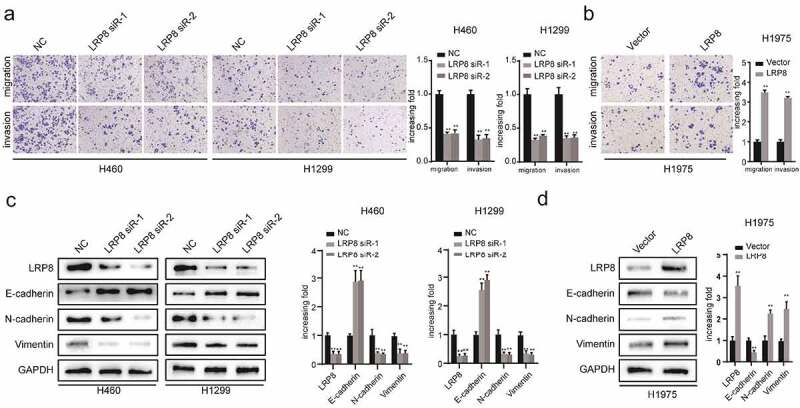
LRP8 enhanced the metastasis of NSCLC cells. (a) Transwell analysis was performed to compare the metastasis potential in the LRP8 knockdown group. (b) H1975 cells migration and invasion capability were detected by transwell assays. The expression of proteins related to metastasis in H1299 and H460 cells (c) and H1975 cells (d) were detected by Western blotting. **p < 0.01. At least three replicate experiments were performed, and the final results were presented as mean ± SD.

We performed transwell assays to evaluate the role of LRP8 in the migration and invasion of NSCLC cells. Suppressing LRP8 decreased the migration and invasion abilities of both H460 and H1299 cells (Figure 3(a)). In contrast, enhanced expression of LRP8 was accompanied by increased migration and invasion of H1975 cells (Figure 3(b)). Concordantly, Western blotting revealed the downregulation of N-cadherin and vimentin and upregulation of E-cadherin in the LRP8-depleted H460 and H1299 cells (Figure 3(c)). However, ectopically elevated LRP8 expression elicited the opposite effects (Figure 3(d)).

### LRP8 regulated the development and metastasis of NSCLC via the Wnt/β-catenin signaling pathway

**Figure 4.**
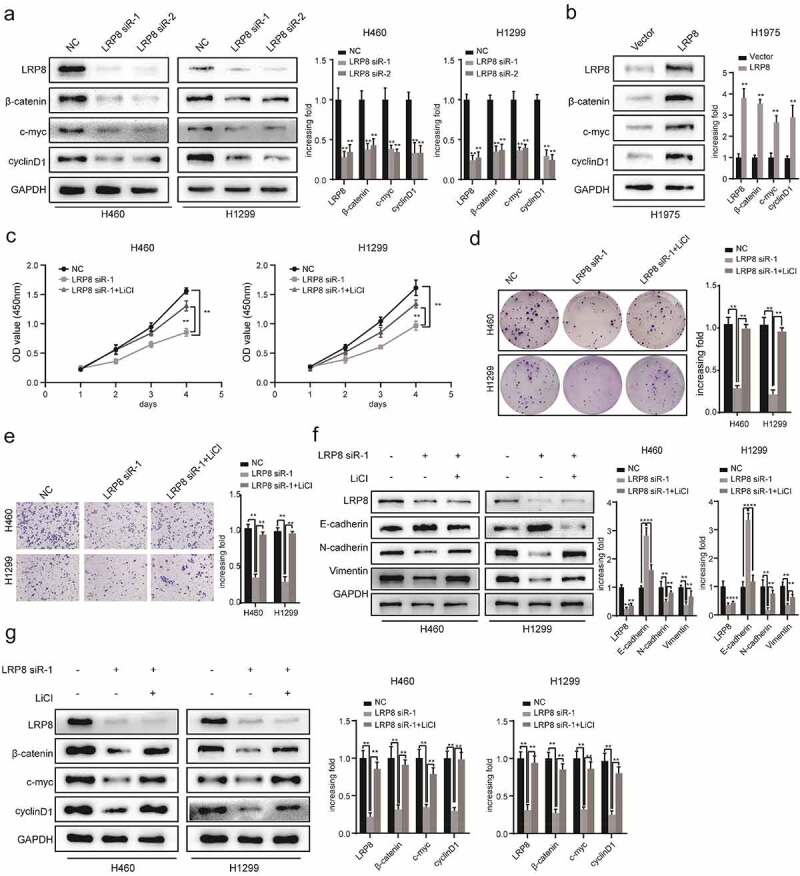
LRP8 motivated NSCLC cells development and metastasis via the Wnt/β-catenin signaling pathway. (a) Expression of Wnt/β-catenin signaling components after silencing LRP8 as detected by Western blotting assays. (b) Expression of Wnt/β-catenin signaling-related factors in overexpression LRP8 group of H1975 cells. CCK-8 assay (c) and colony formation analysis (d) were carried out to evaluate the proliferation abilities of H1299 and H460 cells transfected with LRP8 siRNA or negative vector or LiCl and LRP8 siRNA. (e) Invasion and migration of H1299 and H460 cells after LRP8 downregulation and LiCl addition as detected by Transwell assay. (f) Western blotting analysis for E-cadherin, Vimentin, and N-cadherin to detect the effect of LiCl in LRP8 knockdown. (g) Western blotting assays were performed to elaborate the role of LiCl in Wnt/β-catenin signaling-related factors induced by LRP8 silencing. **p < 0.01. Each experiment was repeated in three independent trials, and mean ± SD was used to describe the results.

Previous studies have reported that dysregulation of the Wnt/β-catenin signaling pathway is responsible for NSCLC tumorigenesis and progression [29,30]. Moreover, Kureel et al. [31] illustrated that LRP8 could increase osteoblast differentiation by activating the Wnt/β-catenin pathway. However, the correlation between LRP8 and the Wnt/β-catenin signaling pathway in NSCLC cells remains to be elucidated. As clearly illustrated in Figure 4(a), LRP8 deficiency reduced the downstream molecules of the Wnt/β-catenin pathway, including β-catenin, c-Myc, and cyclin D1, in H460 and H1299 cells. Conversely, overexpression of LRP8 dramatically elevated the levels of core components of the Wnt/β-catenin pathway in H1975 cells (Figure 4(b)). Hence, LRP8 positively modulated the Wnt/β-catenin signaling pathway. To further investigate whether LRP8 affected NSCLC cell proliferation and metastasis via the Wnt/β-catenin pathway, we used lithium chloride (LiCl), an activator of Wnt signaling [32]. The CCK-8 assay showed that the proliferation of H460 and H1299 cells treated with LRP8 siRNA together with LiCl were partly rescued compared to the cells treated with the LRP8 siRNA group alone (Figure 4(c)). Similarly, colony formation analysis revealed that the decline in cell viability induced by LRP8 knockdown was partially rescued by LiCl (Figure 4(d)). In addition, the reduced migration and invasion caused by LRP8 silencing could be partially abrogated by LiCl treatment in NSCLC cells (Figure 4(e)). Furthermore, the decrease of N-cadherin and vimentin and the increase of E-cadherin in the LRP8 siRNA group of H460 and H1299 cells were reversed by LiCl (Figure 4(f)). Interestingly, we also observed that changes in Wnt/β-catenin pathway-related proteins (β-catenin, c-Myc, and cyclin D1) caused by LRP8 knockdown could be recovered by LiCl (Figure 4(g)).

### Knockdown of LRP8 inhibited tumor growth in vivo

**Figure 5.**
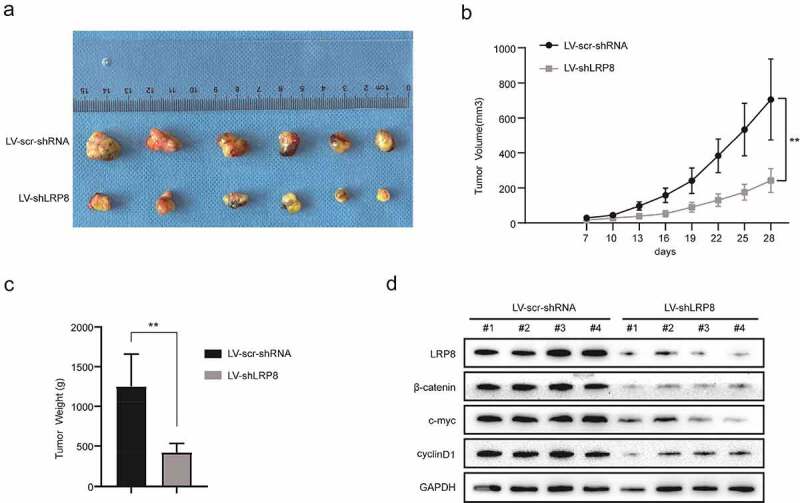
Knockdown of LRP8 inhibited tumor growth *in vivo*. (a) The pictures of nude mice injected with Lv-sh-LRP8 and corresponding control and the tumors formed after 28-day feeding. (b–c) Tumor volumes and weights were calculated between the two groups. (d) Western blotting experiments detecting the expression of Wnt/β-catenin signaling components and LRP8 expression in subcutaneous tumors. **p < 0.01. Three independent trials in each experiment were needed, and the data were presented as mean ± SD.

To evaluate the role of LRP8 *in vivo*, a nude mouse xenograft model was established. H460 cells transfected with Lv-sh-LRP8 or scramble negative control were amplified and injected subcutaneously into the left armpits of nude mice. The tumor mass was obtained carefully and imaged after a 28-day feed (Figure 5(a)). The volume and weight of tumors in the Lv-sh-LRP8 group were lower than those in the control group (Figures 5(b) and (c)). Additionally, compared to the control group, the expression of β-catenin, c-Myc, and cyclin D1 was reduced in the tumors obtained from the LRP8 knockdown group (Figure 5(d)). In summary, LRP8 promoted tumor growth by modulating Wnt/β-catenin signaling *in vivo*, consistent with the *in vitro* results.

## Discussion

In this study, we explored the effects of LRP8 on NSCLC and its underlying molecular mechanisms. LRP8 was overexpressed and correlated with disease prognosis in patients with NSCLC. Moreover, LRP8 facilitated cell proliferation and invasion by activating the Wnt/β-catenin pathway. Therefore, LRP8 serves as a positive regulator of the Wnt/β-catenin signaling pathway, thereby representing a potential therapeutic target for NSCLC treatment.

Previous studies have reported that LDLR greatly contributes to the development of several human cancers [8,33]. In early 2014, Deng et al. [34] confirmed a positive association between LRP6 and NSCLC among the Chinese population via time-of-flight mass spectrometry. Subsequently, the critical role of LRP5/6 in lung cancer progression has also been documented [35,36]. Consistent with the above observations of the Wnt signaling coreceptor LRP5/6, LRP8 was also identified as a target of miR-30b-5p in lung cancer progression and cisplatin resistance [37]. Nevertheless, few studies have described the biological functions and mechanisms of LRP8 in NSCLC. However, in contrast to those studies [38], this is the first study to identify that a higher LRP8 expression significantly correlated with the poor prognosis of NSCLC patients. Moreover, LRP8 silencing suppressed cell proliferation and invasion of NSCLC cells, further implying that LRP8 is a candidate oncogene in NSCLC patients.

Numerous studies have demonstrated that the Wnt/β-catenin signaling pathway is responsible for the migration and metastasis of cancer cells, mediated by the estrogen receptor/NOD-like receptor-LRP8 axis in colon cancer [39,40]. Further, LRP8 depletion reduces breast cancer stem cells and inhibits EMT by regulating the Wnt/β-catenin signaling pathway in triple-negative breast cancer [21]. Therefore, we speculated that LRP8 might play a profound role in NSCLC cells by regulating the Wnt/β-catenin signaling. Mechanistically, our data demonstrated that silencing LRP8 attenuated cell proliferation, invasion, and EMT via the Wnt/β-catenin signaling. In contrast, enhancing LRP8 expression exerted the opposite effect. More intriguingly, rescue assays performed using LiCl, a Wnt pathway activator, showed that LiCl partially rescued the weakened cell proliferation, invasion, and migration induced by LRP8 knockdown. This result agrees with previous studies that have reported that activated Wnt/β-catenin signaling facilitates tumor growth and E-cadherin production in meningioma development and lung cancer [39]. In summary, our data revealed an oncogenic role of LRP8 in NSCLC by orchestrating the Wnt/β-catenin signaling.

However, the exact mechanisms by which LRP8 modulates Wnt/β-catenin signaling remain unknown. LRP8 stimulates Wnt/β-catenin signaling by suppressing Axin2 transcription, thereby controlling osteoblast differentiation [20]. Moreover, LRP8 triggers the phosphorylation of the cytoplasmic adaptor protein Disabled-1 to inactivate glycogen kinase 3-beta, subsequently activating the Wnt/β-catenin pathway [41,42]. Thus, we hypothesize that LRP8 may promote the activation of Wnt signaling via different regulatory mechanisms. Future studies are needed to unravel the exact mechanisms of how LRP8 regulates the Wnt/β-catenin signaling in NSCLC.

## Conclusion

In conclusion, our study provides evidence that LRP8 contributes to NSCLC cell proliferation and invasion by orchestrating the Wnt/β-catenin signaling pathway. Our results highlight that the LRP8-Wnt/β-catenin axis could be a potential prognostic avenue for NSCLC therapy.

## Data Availability

The datasets used or analyzed in this study are available from the corresponding author Ziling Fang upon reasonable request.

## Highlights

- LRP8 is upregulated in NSCLC cells.
- LRP8 significantly correlates with cancer progression in NSCLC patients.
- LRP8 promotes cell proliferation, invasion, and EMT of NSCLC cells.

LRP8 functions as an oncogene by activating the Wnt/β-catenin pathway.
